# Incorporating Patient Input into the Target Product Profile

**DOI:** 10.1007/s43441-025-00783-1

**Published:** 2025-04-25

**Authors:** Jean-Noel Talabardon, Janet E. Church, Masanori Okuse, Michaela Dinboeck, Sheila Dickinson, Simon Messner, Michael Boisclair, Jean-Pierre Malkowski

**Affiliations:** 1 Novartis Pharma AG, Asklepios Building – 5th Floor, Novartis Campus, 4056 Basel, Switzerland; 2 The Sjögren’s Foundation, Reston, VA USA; 3 The Japanese Association for Public Awareness of Psoriasis, Yokohama, Japan; 4 Biomedical Research, Novartis, Cambridge, MA USA

**Keywords:** TPP, Target product profile, Patient input, Patient engagement

## Abstract

Target product profiles (TPP) are summaries of characteristics which drug developers expect to be necessary for a product to meet patients’ needs, receive regulatory and payer approval, and differ from existing treatment options. As the experts on their own disease, patients bring invaluable perspectives to drug development, which cannot be obtained by other means. This communication reports on the development of a systematic guidance framework for a patient-focused, standardized TPP. The guidance was developed in a long-term iterative process, with crucial aspects reviewed and validated with the patient community. Five focus areas of a TPP were identified where patient perspectives are fundamental: target population, unmet need, dosage frequency and route of administration, efficacy endpoints, and acceptability of benefit/risk profile trade-offs. A guiding principle should be to incorporate patient perspectives in a systematic process starting as early as possible. A number of tools are available for obtaining patient perspectives, e.g., desk research, patient advisory boards/patient councils/online bulletin boards, focus groups with patients/caregivers, and/or in-depth interviews. When discussing the proposed process with patient representatives, they identified several key requirements for the interaction between R&D organizations and patient representatives. These include the use of clear language, respect for patients, engagement with patient experts, provision of adequate context and background information. We further discuss the relative importance of integrating patient perspectives into the different focus areas and touch upon the potential benefits to patient organizations from adapting these concepts and processes to enhance their voices in drug development.

## Introduction

Innovation is a crucial driver of improved health outcomes and stronger healthcare systems [1]. For a new therapy to be valuable, it must deliver outcomes that meet patients’, carers’, regulators’ and healthcare providers’ needs at a cost and/or insurance coverage that is acceptable to society. At the same time, the economic incentive needs to be significant enough for commercial stakeholders to risk investing in research and development (R&D) of novel, effective treatments, and device therapies.

To help achieve these manifold objectives, healthcare researchers should engage patients, as the ultimate beneficiaries of healthcare, as active partners contributing their unique experiences. Regulatory organizations such as the US Food and Drug Administration (FDA), the European Medicines Agency (EMA), and the Japanese Pharmaceuticals and Medical Devices Agency (PMDA) have long advocated an integrated patient perspective in different regulatory activities of a medicine’s lifecycle, in order to improve the trust in new approved medicines, and in regulatory decisions [2, 3]. Organizations such as CIOMS (Council for International Organizations of Medical Sciences) have issued recommendations on how to improve patients’ participation in medicines development, matters that ultimately affect their own health [4]. Regulatory agencies and pharmaceutical companies alike recognize and make use of a wide range of evidence beyond randomized clinical trials reflecting patients' experience, such as real-world evidence or registries.

The concept of a Target Product Profile (TPP) was first introduced in 1997 through discussions between the FDA and a Clinical Development working group for improving sponsor and FDA interactions [5, 6]. TPPs are an internal R&D organization tool that formalize what characteristics developers expect to be necessary for a successful medicine, i.e. one that meets the needs of patients, is safe and effective and is widely accessible and ideally reimbursed. By answering the question of “what does success look like?” the TPP is a core strategic tool that can aid the prospective planning of patient-focused approaches and integration of patient input at key milestones [7].

A typical TPP includes important information on target patient population, dosing regimen, pharmaceutical formulation, dosage frequency and route of administration, key efficacy and safety end points, and target effect size, key pricing considerations, and patient access considerations. The existing and/or future standard of care (SoC) is often used as a reference.

Priorities may often differ between the different stakeholders, regulators, payers, physicians, and patients. Patients are the experts on their own disease [8] and this expertise needs to be seen at the same level of seriousness as that of any other stakeholder. To achieve this, the Novartis patient engagement leadership team recognized the need to engage the patient community in this process and shape the TPP template. Among patient-focused aspects in a TPP are indication, route and complexity of administration, meaningful end points, and benefit/risk profile. When asked to review different product profiles, patient experts (patients with knowledge of their disease, often identified and selected by their respective patient organizations) require high-level information on the mode of action to assess the relevance of the new therapy compared with their currently accessible options.

The greater focus on embedding the patient perspective into the drug development matrix has not yet led to the universal implementation of such processes [9]. In a survey from 2019, only two of eleven pharmaceutical companies reported an engagement with patient advocacy groups or patient representatives (patients selected as representatives by a patient organization) in TPP development [10].

Novartis is partnering with the patient community in a growing number of diseases, as the company aims to work towards an enhanced focus on working “with”, not “for” patients to generate patient experience data, evidence, and actionable insights. This stronger commitment is reflected in the development of a guidance about integrating patient perspectives into TPP. The aim of the guidance is to support researchers in reviewing and validating critical aspects of the TPP with the patient community. This communication describes the generation and initial application of this guidance.

## Patient Engagement at Novartis

In 2018 Novartis publicly released The Commitment to Patients and Caregivers [11], which commits the company to respecting and understanding the patient community perspective, conducting responsible clinical trials, expanding access to medicines, and recognizing the importance of transparency and reporting. This includes a commitment to publish progress on how Novartis delivers on these commitments, in response to the expectations of the patient community. The company has employees dedicated to patient engagement/patient advocacy at global, regional, and country levels in a lifecycle model outside the commercial functions in the company. Patient input and engagement across the lifecycle is at the core of the ambition to develop better products faster to meet patients’ needs. This includes creating/co-designing patient relevant endpoints that address outcomes that matter most to patients. Endpoints are the outcomes that are analyzed statistically to determine the efficacy or safety of the therapy being studied. It also includes co-design of clinical trial protocols to enable more rapid enrolment and higher retention rates, and further ensuring that any patient-directed or -facing interface is co-designed with the patient community.

**Figure 1:**
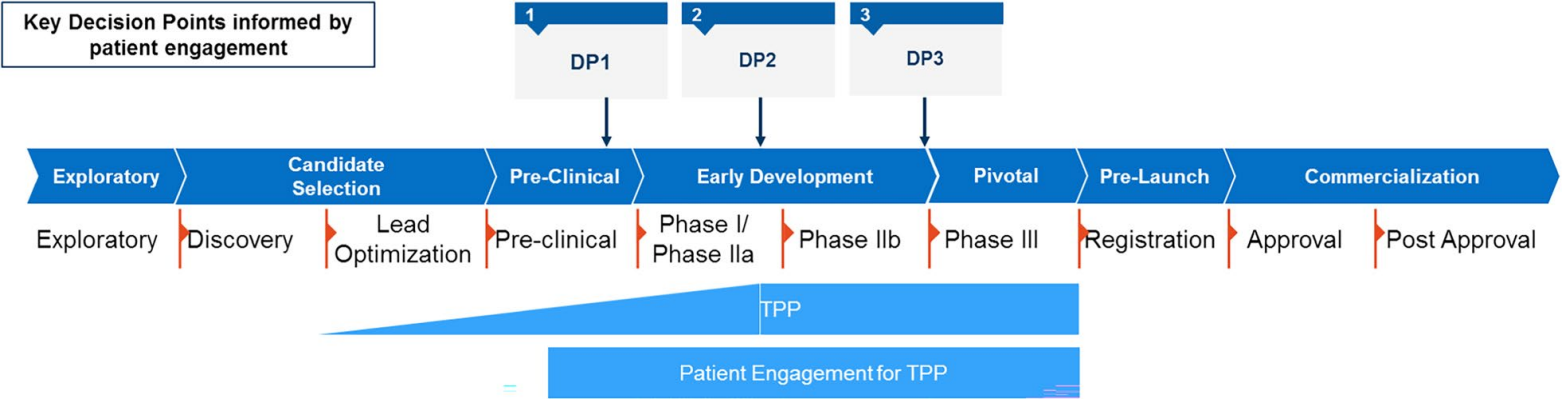
Generic development process with key decision points (DP) reflected in the TPP, and the involvement of patient engagement teams.

To ensure that the patient perspective is consistently and systematically embedded into R&D and commercial decision-making across key milestones, a cross-functional team at Novartis defined key decision points or “tollgates” in a medicine’s lifecycle where the patient perspective must be integrated in order to inform quality patient-focused R&D, and final commercialization decisions (Fig. 1). The TPP is updated at several such decision points, and as necessary depending on what new clinical or pharmaceutical data become available.

## Integrating Patient Perspectives into a TPP

The Novartis standardized patient-focused TPP guidance is the outcome of a long-term iterative process reviewed with patient organization representatives. The patient engagement team and patient community will collaborate on review and validation of the TPP, starting when a compound is in the preclinical phase (Fig. 1).

Whereas patient perspectives are fundamentally relevant to some parts of a TPP, such as those related to the burden of the disease, some sections are usually completed without patient input, e.g., the expected standard of care at time of launch. To ensure patient-informed TPPs, patient experience data is required for five focus areas: target population, unmet need, dosage frequency and route of administration, efficacy endpoints, and acceptability of benefit/risk profile trade-offs. Some of these relate to the lived experience of the disease (patient unmet need, target population, key efficacy endpoints), and some relate to the specific drug product (acceptability of benefit-risk trade-offs, dosage frequency and route of administration). A set of standardized discussion points has been developed, focusing on these five focus areas (Table 1).

**Table 1:** Key Focus Areas for Patient Perspectives Related to the Disease/Indication and Product, Respectively, Guiding Discussions with the Patient Community

Lived experience of disease	Patient lived experience and perspectives on burden of disease
	Patients’ unmet medical need and aims of therapy
	Rationale for choice of key efficacy end point. Relevance to patients of measurement scale and time point of end points
	Target population
Patient perspectives related to product	Dosage frequency and route of administration
	Aspiration, product value
	Patients' views on acceptability of benefit-risk trade-offs, obtained from patient experience data and patient preference studies

Patient perspectives which shape a TPP are obtained in an iterative process, starting as early as possible. The first step, “Desk research”, typically focuses on the disease in general and can usually be taken without resources from patients. Questions which inform the TPP are addressed by accessing available knowledge internally (e.g., patient insights from the Novartis archive, social media listening and literature) and key sources such as FDA voice-of-the-patient reports, the FDA Clinical Outcome Assessment Compendium [12], and the COMET (Core Outcome Measures in Effectiveness Trials) initiative database of core outcome sets [13]. In addition to target literature reviews, desk research further draws on existing information from health technology assessment bodies.

After a gap analysis, the teams engage actively with patient experts to gather additional information and insights to ensure optimal alignment between the TPP and stakeholder needs. Direct interaction with patients is needed in particular for those five focus areas related to the product. Interactions can be on digital platforms or in-person meetings. A number of tools are available to gather patient perspectives on draft TPPs, providing qualitative or quantitative information at varying levels of detail and representativeness (Table 2) [14]. Each tool has strengths and weaknesses as has been described in guidance documents [15]. The preferred method will depend on the questions that need research, and on limiting factors such as budget, time or practical constraints [14].

**Table 2:** Tools for Collecting Patient Perspectives from Different Sources

Tool for obtaining patient perspectives	Description
Desk research	Accessing knowledge available internally and obtainable through literature searches, information from health-technology assessment, and regulatory bodies, social-media listening, and other methods
Patient Advisory Board/Patient Council/Online Bulletin Board	Continuous and regular exchange on topics of common interest to understand the patient community perspective and to seek out, listen, and generate actionable insights
Focus group with patients/caregivers	A qualitative group discussion between participants about a specific topic/project or material, which is typically conducted in-person and moderated by an independent facilitator with special expertise (e.g., researcher, psychologist)
In-depth interviews	Qualitative research that involves conducting intensive individual interviews by moderators with a small number of respondents to explore their perspectives on a particular idea, program or situation

Whichever tool is used to source patient perspectives, patient representatives need to be presented with clear, accessible materials from the R&D organization. Patient representatives also need to be sufficiently healthcare-literate to digest the information in the context of their own situation. Novartis aims to use patient-friendly language and appropriate terminology in all documents presented to community representatives. Supporting materials should also be kept as short as possible without losing important information.

A number of specific recommendations from patient representatives were integrated into the guidance process to optimize the collection of patient perspectives:

- **Use clear language:** Lay language should be used, with added graphics if possible. Symptoms should be specified, e.g., “pain” instead of specialist generic terms like “disease activity”. Rather than using the term “endpoint”, the targeted symptoms should be described and the question should be addressed how an improvement in a particular symptom could improve a patient’s life. Corporate and scientific jargon should be avoided wherever possible and if unavoidable, be explained.
- **Show respect to patients:** The use of more respectful expressions, e.g., “patient” or "people living with the disease" instead of “subject” makes for patient-friendly and accessible communication. Patients should also be addressed as complete beings, not as assemblages of body parts.
- **Solicit advice from patient experts:** Input on a TPP will be most valuable to all stakeholders if consulted patients possess a certain level of knowledge about their disease and relevant treatment methods, and are able to speak on behalf of their patient communities.
- **Provide context:** Specifications such as “moderate to severe” disease or “stages” for oncological indications make it easier for patients to interpret the information.
- **Include mode of action in TPP for new compounds, where possible and necessary**: If a new product presents with a new mode of action, general information on the topic may be relevant to patients, especially if currently available treatments have high non-response rates. This needs to be carefully assessed considering the new product and the existing treatment landscape. To avoid any risk of unlicensed promotion the description must be as limited as possible, for example limited to the drug class (a group of medications and other compounds that have similar chemical structures and a similar mechanism of action, i.e., binding to the same biological target). Descriptions or animations should be accessible; detailed scientific descriptions of mode of action are rarely relevant to patients.
- **Allow questions on trade-offs between different options to understand the acceptability of benefit-risk trade-offs:** Providing a choice between different desirables or risk profiles can help patient experts prioritize characteristics. Useful information can be obtained by adding concrete examples that match the disease and the particular setting, e.g. “would you prefer a drug that may be associated with severe infections or one associated with the side effects of steroids, like blood pressure increase?”. If the acceptability of the benefit-risk profile is not straightforward (e.g. a product profile with strong benefits but also rare, but serious, side-effects), results from qualitative research may need to be followed up and solidified with quantifiable input.

## Case Examples

Below we present examples from Novartis and other R&D organizations of how patient perspectives have been obtained at different stages of the R&D process and were/could be incorporated into the respective key focus areas discussed above. The first two examples illustrate the active engagement by Novartis with patient experts; the following three show how information available via desk research can inform a TPP.

### Target Population: Food Allergy

To explore patients’ perspectives on how the target population for food-allergy treatment may vary between disease types and age groups, Novartis organized a council with patient representatives. Council members reviewed five TPPs for candidate drugs and were asked which target population (defined by disease stage and age group) would be best suited to which drug profile and why. The summary (Table 3) shows how the patient council suggested that target populations for the different hypothetical drug profiles were best defined by age-group (and not by disease type). Moreover, a clearer understanding was required of the cut-off ages to define adolescents and adults. The results helped developers focus on the favored TPPs.

**Table 3:**
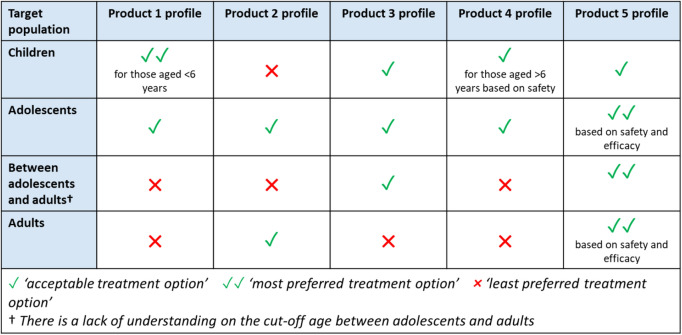
Summary of Patients’ Perspectives on Five Different TPPs for Treatment of Food Allergies, Grouped According to Age of the Possible Target Populations

### Unmet Need: Dry Eye Disease

An advisory board was conducted with patient experts living with a dry eye disease and/or chronic ocular surface pain [16]. The aim was to gather perspectives on how current and hypothetical future treatment landscapes meet patients’ needs, and what characteristics patients consider important in a medicine. The need for more convenient treatments came out strongly, with twice-daily dosing and reduced or eliminated need for artificial tear products viewed as highly desirable characteristics compared with other available options. A new treatment for ocular surface pain would be particularly welcomed. In addition to the input on TPP, participants highlighted the possible help from patient organizations, such as the Sjögren’s Foundation in the US, for clinical trial recruitment of patients living with Sjögren's, as dry eye is one common symptom in these patients.

### Dosage Frequency and Route of Administration: Diffuse Large B-Cell Lymphoma (DLBCL) and Follicular Lymphoma (FL)

This example relates to the need to support the registration of a new formulation of an existing drug. To obtain patient perspectives on the preferred mode of administration for a specific drug (rituximab) for DLBCL and FL, a randomized, open-label, crossover study was designed. The primary endpoint was overall preference for rituximab administered intravenously or subcutaneously, as expressed in a Patient Preference Questionnaire. Patients graded their preferences as very strong, fairly strong, or not very strong. Most patients preferred subcutaneous administration, mainly because of shorter administration time and less discomfort [17]. This information could be relevant for the development of patient-focused TPPs for future drugs intended for the same or similar disease areas.

### Endpoints: Chronic Myeloid Leukemia

TPPs and clinical trial endpoints need to reflect the outcomes that matter most to patients. For chronic myeloid leukemia, there is a need for specific patient-reported questionnaires to assess health-related quality of life (HRQoL). To develop such a tool, a large number of patients including the CML Advocates Network were included in a predefined and systematic, international, iterative process [18]. The resulting EORTC QLQ-CML24 questionnaire includes 24 items and identifies various areas of concern ranging from a key set of symptoms to more general aspects, such as satisfaction with social life. The questionnaire is an important tool when developing TPPs and patient-relevant clinical trial endpoints in chronic myeloid leukemia.

### Benefit/Risk Profile: Alopecia Areata

Alopecia areata (AA) is an autoimmune disease that causes hair loss. Effective treatment with Janus kinase (Jak) inhibitors is emerging, but the agents are associated with increased risk of serious infections, malignancies, and thromboembolic events. A TPP for an alopecia medicine should include patients’ views on the trade-offs they may be willing to make between benefits and risks to inform regulatory decision-making. However, such information was not captured in published surveys of patient preferences [19]. Pfizer designed an online discrete-choice experiment, based on in-depth qualitative patient interviews, a literature review, and consultation with clinical experts [20]. The survey focused on people diagnosed with severe AA. The most important attribute for both adult and adolescent respondents was a 50% probability of hair regrowth on most of the scalp. Adults were concerned about the risks of serious infection, cancer, and blood clots, whereas adolescents were only concerned about the risk of cancer. This information about patients’ views on the acceptability of benefit-risk trade-offs, obtainable by desk research, is highly valuable for the development of patient-focused TPPs for future drugs intended for alopecia areata.

## Discussion

In today’s medicines development landscape, patient experience data are a pre-requisite for a regulatory submission [21]. As the experts on their own disease, patients bring an invaluable perspective to drug development, which cannot be obtained by other means. The present communication describes to our best knowledge the first systematic guidance framework for development of a patient-focused, standardized TPP with crucial aspects reviewed and validated with the patient community. With appropriate modifications to meet different conditions, we believe this guidance can be leveraged across the medicines development community.

### Get the Timing Right

The TPP development process we describe embeds patient experiences, preferences and recommendations from the patient community starting early in development. This is important in order to ensure that full development uses patient-relevant endpoints and label claims. “Sanity checks” during the exploratory and lead-optimization phases may help weed out unsuitable candidate molecules early. In practice, resources are a limiting factor for all R&D organizations and also for patients. A guiding principle is to incorporate patient perspectives in a systematic process starting as early as possible.

### Focus Areas for Patient Perspectives

This TPP development process emphasizes the value of patient perspectives on five topics: target population, unmet need, dosage frequency and route of administration, efficacy endpoints, and acceptability of benefit/​risk profile trade-off considerations for new therapies. Table 4 provides examples of the added value from including patient perspectives on these five areas in a TPP. Other ultimate benefits from patient engagement in R&D include enhanced clinical study recruitment and retention, better adherence, and a stronger focus on health equity [22–24].

**Table 4:** Added Value from Including Patient Perspectives in Five Focus Areas in a TPP.

Focus area	Examples of added value of integrated patient perspectives
Target population	Identification of potential heterogeneity in the target population Pinpointing possible subgroups relevant to the drug
Unmet need	Better understanding of what is needed from a new therapy in the light of patients’ perspectives on the efficacy, side-effects and/or mode of administration of current therapies
Dosage frequency and route of administration	Within the technical limitations, patients’ needs should be accommodated as far as possible
Efficacy endpoints	Successful phase 3 trials which use patient-relevant endpoints (if accepted by regulators) will drive the development of medications which target the needs that matter most to patients
Benefit/risk profile trade-offs	Patient perspectives on acceptability of benefit-risk trade-offs are particularly important if: (a) A new drug is expected to offer substantial benefit together with rare but serious risks (b) A new drug is expected to offer very modest benefit and minimal risks compared with current alternatives

Arguably, efficacy endpoints are the most important of the five topics. If a TPP includes patient-relevant endpoints (accepted by regulators) in registration trials, a drug which has gone through successful clinical development can be launched with patient-relevant label claims. This beneficial outcome may be complicated to achieve. To incorporate patients’ perspective on the relevance and burden of specific symptoms is likely to be fairly straightforward. The identification of appropriate endpoints to measure such symptoms will need discussions involving many different disciplines and stakeholders including regulatory bodies, yet, difficulties should not diminish the importance of this topic. The growing use of Core Outcome Sets offers an encouraging path forward. A core outcome set is a minimum set of outcomes that should be measured and reported in all clinical trials undertaken in a specific health condition [25]. Core Outcome Sets are developed with patients and carers included in the process to identify patient-relevant outcomes [26].

### Need to Adapt

The framework presented here relies on close multidisciplinary collaboration and will require multiple stakeholders to consider how to implement its components into existing processes. R&D organizations will need to ensure that adequate human and financial resources are allocated to the task of successfully integrating patient input into an updated TPP before the next development milestone. For commercial R&D organizations, sufficient time and resources must also be invested in translating a company TPP with its internal jargon into a patient-friendly version. Internal, medical, and scientific vocabulary ensures rapid and specific communication among and between researchers and regulators. Yet patients’ views will only be valuable if advisers have a good understanding of the characteristics and goals formalized in a TPP. Conversely, patients’ views, expressed in lay language, need to translate back carefully into developer terminology for internal processes to properly reflect patients' perspectives. This two-way information exchange will require strong internal and external communication skills.

### Use Many Resources of Patient-Experience Information

For a commercial R&D organization, information about patient experiences, needs and priorities is acquired through many channels. There is a risk that resources are spent unnecessarily on developing new engagement with patients when relevant, up-to-date information is already available from existing sources. It is helpful to research such data and identify remaining questions before engaging with patients, to ensure relevant questions are asked and relevant information is provided by patient representatives.

Information relevant to patient engagement is to a large extent qualitative and unstructured. There is great interest in developing new or repurposing existing methods such as natural-language programming to expand the capacity of qualitative researchers to analyze larger amounts and more diverse data types. These methods can increase sample size, reduce project time, and significantly reduce costs. Standardization of methods will allow new data to be analyzed using the same interpretation and identification [27]. Such developments could further enhance the quality and reliability of patient-derived insights in the future.

### Outlook

The implementation of patient-focused, standardized TPPs with key aspects validated with the patient community brings a number of potential benefits: increased patient-focused relevance, closer alignment with regulatory expectations, and labels which include patient-relevant endpoints. In our experience, the patient community has been appreciative of their participation in these processes, reporting an improved understanding of their lived experience and a potential for their input to have greater influence on the aspirational profile of a drug in development. In a wider perspective, patient engagement can lead to enhanced clinical study recruitment and retention, and a stronger focus on diversity, health equity and access to treatments. In addition, clinical adoption of new treatments may be increased if claims and associated communications are tailored closer to those needs that matter most to patients.

There have been doubts about the degree of awareness of the importance of a more evolved patient voice among rank-and-file drug reviewers and pharmaceutical decision makers [28]. The development of patient-focused, standardized TPPs represents a route towards more widespread awareness among those closely involved in the development processes.

With time, the process we describe will provide the patient community with greater experience of the full process from providing patient input into the early TPP to seeing the drug come to market. The formalization of “what does success look like” for a specific drug in a TPP and the focus on five areas of relevance may provide clarity on the information R&D organizations use in drug development. Such knowledge would help patient organizations focus their communications to enhance their influence with R&D organizations.

There have recently been calls for the development of “Patient experience data dossiers” which would be owned by patient organizations and would consolidate evidence from various sources, including diagnosis experiences, clinical and economic aspects of the disease, and perspectives on available treatments and unmet needs [29, 30]. Such dossiers would be aids to specific and meaningful discussions between patients and numerous healthcare stakeholders, and could be a useful resource for any organization seeking to develop a patient-informed TPP.

In conclusion, the push to integrate patient lived experiences and needs into the TPP and to do this as early as possible has the potential to enhance the medicine development process and provide additional value to all stakeholders. Over the coming years we hope a greater experience with the process in our and other R&D organizations will provide useful tweaks and adaptations to optimize the TPP guidance framework.

## Data Availability

No datasets were generated or analysed during the current study.

## References

[1] Flessa S, Huebner C. Innovations in Health Care—a conceptual framework. Int J Environ Res Public Health. 2021;18:10026. 34639328 10.3390/ijerph181910026 PMC8508443

[2] European Medicines Agency. Engagement framework: EMA and patients, consumers and their organisations [Internet]. 2022. Available from: https://www.ema.europa.eu/documents/other/engagement-framework-european-medicines-agency-patients-consumers-their-organisations_en.pdf

[3] US Food and Drug Administration. FDA Patient-Focused Drug Development Guidance Series for Enhancing the Incorporation of the Patient’s Voice in Medical Product Development and Regulatory Decision Making. FDA [Internet]. 2023 [cited 2023 Oct 30]; Available from: https://www.fda.gov/drugs/development-approval-process-drugs/fda-patient-focused-drug-development-guidance-series-enhancing-incorporation-patients-voice-medical

[4] CIOMS Working Group XI on Patient involvement in the development, regulation and safe use of medicines. Patient involvement in the development, regulation and safe use of medicines [Internet]. Council for International Organizations of Medical Sciences (CIOMS); 2022. Available from: https://cioms.ch/publications/product/patient-involvement/

[5] Bandyopadhyay A. Target Product Profile: A Planning Tool for the Drug Development. MOJ Bioequivalence Bioavailab [Internet]. 2017 [cited 2024 Feb 10];3. Available from: https://medcraveonline.com/MOJBB/target-product-profile-a-planning-tool-for-the-drug-development.html

[6] World Health Organisation. Target product profiles [Internet]. [cited 2024 Apr 17]. Available from: https://www.who.int/observatories/global-observatory-on-health-research-and-development/analyses-and-syntheses/target-product-profile/who-target-product-profiles

[7] Algorri M, Cauchon NS, Christian T, O’Connell C, Vaidya P. Patient-centric product development: a summary of select regulatory CMC and device considerations. J Pharm Sci. 2023;112:922–36. 36739904 10.1016/j.xphs.2023.01.029

[8] Patient Engagement for Medicines Development. The PFMD Book of Good Practices [Internet]. 2020 [cited 2025 Feb 26]. Available from: https://patientfocusedmedicine.org/bogp/2020/the-book-of-good-practices.pdf

[9] Dinboeck M, Noopur N, Krause A, Kaur S. Patient Involvement in Regulatory and Health Technology Assessment Processes: A Call for Enhanced Alignment. Value Outcomes Spotlight. 2024;27–33.

[10] Wang T, McAuslane N, Goettsch WG, Leufkens HGM, De Bruin ML. Challenges and opportunities for companies to build HTA/payer perspectives into drug development through the use of a dynamic target product profile. Front Pharmacol. 2022;13: 948161. 35924050 10.3389/fphar.2022.948161 PMC9340272

[11] Novartis AG. Novartis commitment to patients and caregivers 2025 factsheet [Internet]. 2025 [cited 2025 Mar 1]. Available from: https://www.novartis.com/sites/novartis_com/files/novartis-commitment-patients-caregivers-factsheet.pdf

[12] U.S. Food and Drug Administration. Clinical Outcome Assessment Compendium. FDA [Internet]. 2021 [cited 2024 Apr 16]; Available from: https://www.fda.gov/media/130138/download?attachment

[13] COMET Initiative [Internet]. [cited 2024 May 5]. Available from: https://www.comet-initiative.org/

[14] US Food and Drug Administration. Patient-Focused Drug Development: Collecting Comprehensive and Representative Input [Internet]. US Food and Drug Administration; 2020 [cited 2024 Feb 21]. Available from: https://www.fda.gov/drugs/guidance-compliance-regulatory-information/guidances-drugs

[15] US Food and Drug Administration. Patient-Focused Drug Development: Methods to Identify What Is Important to Patients [Internet]. US Food and Drug Administration; 2022 [cited 2024 Feb 21]. Available from: https://www.fda.gov/regulatory-information/search-fda-guidance-documents/patient-focused-drug-development-methods-identify-what-important-patients

[16] Caffery B, Petris R, Hammitt KM, Montecchi-Palmer M, Haque S, Malkowski J-P, et al. Patient perspectives on dry eye disease and chronic ocular surface pain: Insights from a virtual community-moderated dialogue. Eur J Ophthalmol. 2022;11206721221125263. 10.1177/11206721221125263 36071618

[17] Rummel M, Kim TM, Plenteda C, Capochiani E, Mendoza M, Smith R, et al. Prefmab: final analysis of patient preference for subcutaneous versus intravenous rituximab in previously untreated CD20+ diffuse large B-cell lymphoma and follicular lymphoma. Blood. 2015;126:3972–3972.

[18] Efficace F, Baccarani M, Breccia M, Saussele S, Abel G, Caocci G, et al. International development of an EORTC questionnaire for assessing health-related quality of life in chronic myeloid leukemia patients: the EORTC QLQ-CML24. Qual Life Res. 2014;23:825–36. 24026634 10.1007/s11136-013-0523-5

[19] O’Connor LF, Wells KM. Characterizing the willingness to undergo treatment in patients with alopecia areata. Arch Dermatol Res. 2022;314:749–57. 34609599 10.1007/s00403-021-02286-z

[20] Tervonen T, Whichello C, Law E, Mauer J, Mitra D, Trapali M, et al. Treatment preferences of adults and adolescents with alopecia areata: a discrete choice experiment. J Dermatol. 2024;51:243–52. 38087841 10.1111/1346-8138.17056 PMC11483896

[21] US Food and Drug Administration. Assessment of the Use of Patient Experience Data in Regulatory Decision-Making [Internet]. 2021 [cited 2025 Mar 1]. Available from: https://www.fda.gov/media/150405/download?attachment

[22] Frank L, Forsythe L, Ellis L, Schrandt S, Sheridan S, Gerson J, et al. Conceptual and practical foundations of patient engagement in research at the patient-centered outcomes research institute. Qual Life Res. 2015;24:1033–41. 25560774 10.1007/s11136-014-0893-3 PMC4412554

[23] Merker VL, Hyde JK, Herbst A, Solch AK, Mohr DC, Gaj L, et al. Evaluating the impacts of patient engagement on health services research teams: lessons from the veteran consulting network. J Gen Intern Med. 2022;37:33–41. 35349028 10.1007/s11606-021-06987-z PMC8993982

[24] Zickmund SL, Frosch DL, Carman KL. Patient and veteran engagement in health research: the emergence of a field of study. J Gen Intern Med. 2022;37:3–5. 35347562 10.1007/s11606-022-07393-9 PMC8993997

[25] Kirkham JJ, Williamson P. Core outcome sets in medical research. BMJ Med. 2022;1: e000284. 36936568 10.1136/bmjmed-2022-000284 PMC9951367

[26] Kirkham JJ, Davis K, Altman DG, Blazeby JM, Clarke M, Tunis S, et al. Core outcome set-STAndards for development: the COS-STAD recommendations. PLoS Med. 2017;14: e1002447. 29145404 10.1371/journal.pmed.1002447 PMC5689835

[27] Abram MD, Mancini KT, Parker RD. Methods to integrate natural language processing into qualitative research. Int J Qual Methods. 2020;19:160940692098460.

[28] Pitts PJ. Towards meaningful engagement for the patient voice. Patient - Patient-Centered Outcomes Res. 2019;12:361–3. 10.1007/s40271-019-00366-x 31165399

[29] Oehrlein EM, Edward HA, Howarth TJ, Vandigo J. Listening sessions can help CMS become more patient-centered. Here’s how the sessions could be more effective. Health Aff (Millwood) [Internet]. 2023 [cited 2024 Apr 25]; Available from: http://www.healthaffairs.org/do/10.1377/forefront.20231031.623114/full/

[30] Witting L. Exploring the Patient Experience Dossier [Internet]. Natl. Health Counc. 2022 [cited 2024 Apr 25]. Available from: https://nationalhealthcouncil.org/blog/exploring-the-patient-experience-dossier/

